# Triglyceride–glucose index and its derivatives in women with polyendocrine metabolic ovarian syndrome: associations with insulin resistance markers

**DOI:** 10.1590/1806-9282.20260826

**Published:** 2026-11-09

**Authors:** Neslişah Gürel Köksal, Yasemin Emür Günay

**Affiliations:** 1 Giresun University, Training and Research Hospital, Department of Family Medicine – Giresun, Turkey.; 2 Giresun University, Training and Research Hospital, Department of Endocrinology and Metabolism – Giresun, Turkey.

**Keywords:** Biomarkers, Insulin resistance, Insulin sensitivity, Metabolic syndrome, Polycystic ovary syndrome

## Abstract

**OBJECTIVE:**

The aim of this study was to compare the triglyceride–glucose index and its derivatives between women with polyendocrine metabolic ovarian syndrome and healthy controls and to examine their associations with established fasting-based surrogate markers of insulin resistance.

**METHODS:**

The final analytical cohort consisted of 109 women with polyendocrine metabolic ovarian syndrome and 91 healthy controls. Homeostasis model assessment of insulin resistance, quantitative insulin sensitivity check index, fasting glucose-to-insulin ratio, triglyceride–glucose index, triglyceride–glucose-body mass index, and triglyceride-to-high-density lipoprotein cholesterol ratio were calculated. Between-group comparisons and correlation analyses were performed, with age, body mass index, and waist circumference considered in adjusted analyses.

**RESULTS:**

Women with polyendocrine metabolic ovarian syndrome had significantly higher homeostasis model assessment of insulin resistance values than healthy controls [median: 3.66 (range: 0.76–37.72) vs. 0.86 (range: 0.30–2.70), p<0.001]. The between-group difference in the triglyceride–glucose index remained significant after adjustment for age, body mass index, and waist circumference (F=20.489, p<0.001, ηp^2^=0.094). Within the polyendocrine metabolic ovarian syndrome group, triglyceride–glucose was positively correlated with homeostasis model assessment of insulin resistance (r=0.505, p<0.001) and negatively correlated with quantitative insulin sensitivity check index (r=-0.505, p<0.001) and fasting glucose-to-insulin ratio (r=-0.449, p<0.001).

**CONCLUSION:**

Triglyceride–glucose and triglyceride–glucose-body mass index were higher in women with polyendocrine metabolic ovarian syndrome than in healthy controls and were associated with fasting-based surrogate measures of insulin resistance. These findings suggest that triglyceride–glucose-based indices may provide complementary information regarding the metabolic profile associated with polyendocrine metabolic ovarian syndrome. Their clinical utility requires further validation in prospective studies using direct measures of insulin sensitivity.

## INTRODUCTION

Polycystic ovary syndrome (PCOS) is a common, heterogeneous endocrine–metabolic disorder affecting women of reproductive age and encompassing reproductive, endocrine, metabolic, and psychological manifestations. Growing recognition that the conventional name disproportionately emphasizes ovarian morphology and may obscure the broader multisystem nature of the condition has prompted reconsideration of its terminology. International stakeholder research demonstrated substantial support among both affected individuals and health professionals for a name that more accurately reflects the endocrine and metabolic dimensions of the syndrome^1,2^. Following a multistep global consensus process, polyendocrine metabolic ovarian syndrome (PMOS) was introduced in 2026 as the new terminology for the condition previously known as PCOS, with the terms "polyendocrine," "metabolic," and "ovarian" selected to better represent its multisystem pathophysiology^1^. This terminology has subsequently been supported within contemporary professional and scientific frameworks, including the European Society of Gynecology, which has emphasized that the transition from PCOS to PMOS represents an evolution in nomenclature rather than the introduction of a new disease entity or a change in the underlying diagnostic framework^3^. Accordingly, the diagnostic criteria used in the present study remain those established for PCOS; participants were identified according to the 2003 Rotterdam criteria. Throughout this manuscript, PMOS is used when referring to the syndrome under the recently adopted terminology, whereas PCOS is retained when referring to the Rotterdam diagnostic criteria and to previous studies conducted and published under the conventional terminology^1-3^.

Insulin resistance (IR) is a central metabolic feature of PMOS and may occur across phenotypes, including in women without overt obesity. Tissue-specific alterations in insulin signaling in skeletal muscle, adipose tissue, and liver, together with compensatory hyperinsulinemia, contribute to systemic metabolic dysfunction and may enhance ovarian androgen production^4,5^. These abnormalities contribute to the increased burden of dyslipidemia, impaired glucose tolerance, type 2 diabetes, and metabolic syndrome observed in affected women^4-6^. Characterizing IR-related metabolic abnormalities is therefore important for understanding cardiometabolic risk and informing metabolic assessment in women with PMOS.

Although the hyperinsulinaemic–euglycaemic clamp is considered a reference method for assessing insulin sensitivity, its cost, complexity, and resource requirements limit its routine clinical use. Consequently, fasting-based surrogate indices, including the homeostasis model assessment of insulin resistance (HOMA-IR), quantitative insulin sensitivity check index (QUICKI), and fasting glucose-to-insulin ratio (FG/IR), are widely used in clinical and epidemiological research. However, these indices require insulin measurement and may be influenced by assay variability, population-specific characteristics, and adiposity^5,7^. Importantly, HOMA-IR and related fasting indices should be regarded as surrogate measures of IR rather than reference standards for direct assessment of insulin sensitivity.

The triglyceride–glucose (TyG) index, derived from fasting triglyceride and glucose concentrations, has emerged as a simple, non-insulin-based metabolic marker associated with IR. Previous studies have demonstrated significant associations between the TyG index and fasting-based surrogate measures of IR and have linked TyG and related indices to metabolic syndrome components, non-alcoholic fatty liver disease, and other adverse metabolic features in women with PCOS^8-10^. Because TyG-derived measures incorporate routinely available metabolic and, in some cases, anthropometric parameters, they may provide complementary information regarding the metabolic profile of affected women. However, direct comparisons of TyG-based indices with multiple conventional fasting-based surrogate markers of IR within the same PMOS cohort remain limited, particularly after accounting for relevant anthropometric factors such as age, body mass index (BMI), and waist circumference.

We hypothesized that the TyG index and TyG-BMI would be higher in women with PMOS than in healthy controls and would show significant associations with fasting-based surrogate measures of IR, including positive associations with HOMA-IR and inverse associations with QUICKI and FG/IR. We further hypothesized that TyG-based indices may provide complementary, non-insulin-based information regarding the metabolic profile associated with PMOS. Therefore, this study aimed to compare the TyG index, TyG-BMI, triglyceride-to-high-density lipoprotein cholesterol (TG/HDL-C) ratio, and conventional fasting-based IR-related indices between women with PMOS and healthy controls and to examine the associations of TyG-based indices with established fasting-based surrogate markers of IR.

## METHODS

### Study design and participant selection

This cross-sectional analytical study was conducted at Giresun University Training and Research Hospital between January 2025 and April 2025. The study protocol was approved by the Scientific Research Ethics Committee of Giresun University Training and Research Hospital (approval date: 11 December 2024; approval no: 11.12.2024/17). Data were collected anonymously without personal identifiers, with confidentiality rigorously maintained. All procedures were performed in accordance with the principles of the Declaration of Helsinki. A large language model (ChatGPT) was used to assist with language editing in early manuscript drafts; all content was checked and verified by the authors.

The analytical cohort comprised 200 women aged 19–42 years: 109 women with PCOS and 91 healthy controls. Women classified under the updated PMOS terminology had originally been diagnosed with PCOS by an endocrinologist according to the 2003 Rotterdam criteria, requiring at least two of the following: oligo-/anovulation, clinical and/or biochemical hyperandrogenism, and polycystic ovarian morphology on ultrasound. Exclusion criteria for both groups were known diabetes mellitus, thyroid dysfunction, hyperprolactinemia, pregnancy, and use of medications affecting glucose or lipid metabolism (e.g., metformin, glucocorticoids, lipid-lowering agents, or hormonal contraceptives) within the preceding 3 months.

### Instrumentation and data collection

Clinical and laboratory data were retrospectively retrieved from the hospital electronic medical records. Anthropometric data included age, BMI, and waist circumference; BMI was calculated as weight divided by height squared when not directly available. Fasting venous blood samples, obtained after at least 8 h of fasting, were analyzed for glucose, insulin, total cholesterol (TC), triglycerides (TG), high-density lipoprotein cholesterol, low-density lipoprotein cholosterol, luteinizing hormone (LH), and follicle-stimulating hormone (FSH). Biochemical measurements were performed in the hospital central laboratory using enzymatic colorimetric assays on an automated analyzer, while insulin, LH, and FSH were measured by chemiluminescence immunoassays.

### Calculation of insulin resistance-related indices

Insulin resistance and insulin sensitivity were evaluated using fasting-based surrogate indices. Glucose and lipid parameters were recorded in mg/dL and insulin in μIU/mL. The following indices were calculated: TyG index=ln[TG×glucose/2]; TyG-BMI=TyG index×BMI; TC/HDL-C=TC/HDL-C; TG/HDL-C=TG/HDL-C; HOMA-IR=fasting glucose×fasting insulin/405; QUICKI=1/[log_10_(fasting insulin)+log_10_(fasting glucose)]; and FG/IR=fasting glucose/fasting insulin. HOMA-IR, QUICKI, and FG/IR were treated as fasting-based surrogate measures rather than reference-standard diagnostic tests.

### Statistical analysis

The normality of continuous variables was assessed using the Kolmogorov-Smirnov test and visual inspection methods, and data were presented as mean±standard deviation, median (minimum–maximum), or frequency and percentage, as appropriate. Between-group comparisons were performed using the independent-samples t-test, Mann-Whitney U test, chi-square test, or Fisher's exact test according to data distribution and variable type. Adjusted group comparisons were conducted using analysis of covariance (ANCOVA) or Quade's non-parametric ANCOVA, with age, BMI, and waist circumference included as covariates. Associations between metabolic indices were evaluated using Spearman and partial correlation analyses. All analyses were performed using IBM SPSS Statistics version 26.0, with p<0.05 accepted as statistically significant. An a priori power analysis was performed using G*Power 3.1 for the comparison of two independent groups. Assuming a two-sided alpha level of 0.05, 95% statistical power, and a medium effect size (Cohen's d=0.51), the required total sample size was 200 participants (109 in the PMOS/PCOS group and 91 controls).

## RESULTS

### Cohort characteristics and metabolic profile

The final analytical cohort consisted of 109 women with PMOS and 91 healthy controls. In unadjusted comparisons, significant between-group differences were observed for the evaluated metabolic and fasting-based IR/sensitivity indices (Table 1). Correctly calculated HOMA-IR values were significantly higher in women with PMOS than in healthy controls [median: 3.66 (range: 0.76–37.72) vs. 0.86 (range: 0.30–2.70), p<0.001].

**Table 1:** Comparison of metabolic and insulin resistance-related indices between control and polyendocrine metabolic ovarian syndrome groups.

Variable	Control (n=91)	PMOS (n=109)	Test statistic	p-value
TyG index	7.97 (7.21–8.73)	8.35 (7.25–9.80)	-5.029	<0.001
HOMA-IR	0.89 (0.30–2.70)	3.35 (0.76–37.72)	-11.632	<0.001
TyG-BMI	197.32 (144.85–313.20)	206.19 (140.38–336.75)	-3.428	0.001
TC/HDL-C ratio	3.79 (2.25–6.20)	3.47 (1.65–8.17)	-2.988	0.003
TG/HDL-C ratio	2.30 (0.85–4.82)	1.76 (0.43–14.10)	-2.305	0.021
QUICKI	0.39±0.03	0.32±0.03	16.180	<0.001
FG/IR	17.85 (6.27–38.41)	5.85 (1.34–20.04)	-10.691	<0.001

Data are presented as median (minimum–maximum) or mean±standard deviation, as appropriate. Mann-Whitney U test was used for all variables except QUICKI, which was analyzed with the independent-samples t-test. HOMA-IR was calculated as fasting glucose (mg/dL)×fasting insulin (μIU/mL)/405. PMOS: polyendocrine metabolic ovarian syndrome; TyG: triglyceride–glucose; BMI: body mass index; TC: total cholesterol; HDL-C: high-density lipoprotein cholesterol; TG: triglycerides; HOMA-IR: homeostasis model assessment of insulin resistance; QUICKI: quantitative insulin sensitivity check index; FG/IR: fasting glucose-to-insulin ratio.

### Adjusted comparisons according to age, body mass index, and waist circumference

Because age, BMI, and waist circumference may influence metabolic indices, adjusted comparisons were performed using these variables as covariates. After adjustment, the between-group difference in the TyG index remained statistically significant (F=20.489, p<0.001, ηp^2^=0.094), indicating that higher TyG values in women with PMOS were not explained solely by age or anthropometric differences (Table 2). Significant adjusted differences also persisted for HOMA-IR, TyG-BMI, QUICKI, and FG/IR, whereas TC/HDL-C was no longer significant and TG/HDL-C showed a small effect size.

**Table 2:** Age-, body mass index, and waist circumference-adjusted comparisons of metabolic indices between control and polyendocrine metabolic ovarian syndrome groups.

Variable	F	p-value	ηp^2^
TyG index	20.489	<0.001	0.094
HOMA-IR	269.112	<0.001	0.576
TyG-BMI	16.641	<0.001	0.078
TC/HDL-C ratio	2.871	0.092	0.014
TG/HDL-C ratio	5.264	0.023	0.026
QUICKI	251.595	<0.001	0.563
FG/IR	166.099	<0.001	0.456

Age, BMI, and waist circumference were included as covariates. Parametric ANCOVA or Quade's non-parametric ANCOVA was applied according to data distribution. Effect sizes are expressed as partial eta squared (ηp^2^). HOMA-IR was recalculated using fasting glucose (mg/dL)×fasting insulin (μIU/mL)/405. PMOS: polyendocrine metabolic ovarian syndrome; BMI: body mass index; TyG: triglyceride–glucose; TC: total cholesterol; HDL-C: high-density lipoprotein cholesterol; TG: triglycerides; HOMA-IR: homeostasis model assessment of insulin resistance; QUICKI: quantitative insulin sensitivity check index; FG/IR: fasting glucose-to-insulin ratio; ANCOVA: analysis of covariance.

### Correlations among triglyceride–glucose-based and conventional fasting-based indices

Within the PMOS group, Spearman correlation analyses showed that the TyG index was moderately and positively correlated with HOMA-IR (r=0.505, p<0.001) and negatively correlated with QUICKI (r=-0.505, p<0.001) and FG/IR (r=-0.449, p<0.001). In partial correlation analyses controlling for age, BMI, and waist circumference, TyG remained positively associated with HOMA-IR (r=0.393, p<0.001), TyG-BMI (r=0.988, p<0.001), TC/HDL-C (r=0.695, p<0.001), and TG/HDL-C (r=0.846, p<0.001), and inversely associated with QUICKI (r=-0.519, p<0.001) and FG/IR (r=-0.454, p<0.001) (Table 3).

**Table 3:** Partial correlations among metabolic indices in women with polyendocrine metabolic ovarian syndrome after adjustment for age, body mass index, and waist circumference.

Variable	TyG index	HOMA-IR	TyG-BMI	TC/HDL-C	TG/HDL-C	QUICKI
HOMA-IR	0.393 (<0.001)					
TyG-BMI	0.988 (<0.001)	0.390 (<0.001)				
TC/HDL-C	0.695 (<0.001)	0.262 (0.007)	0.693 (<0.001)			
TG/HDL-C	0.846 (<0.001)	0.265 (0.006)	0.831 (<0.001)	0.740 (<0.001)		
QUICKI	-0.519 (<0.001)	-0.795 (<0.001)	-0.517 (<0.001)	-0.371 (<0.001)	-0.369 (<0.001)	
FG/IR	-0.454 (<0.001)	-0.623 (<0.001)	-0.449 (<0.001)	-0.340 (<0.001)	-0.354 (<0.001)	0.942 (<0.001)

Cells show partial correlation coefficient r (p-value). Age, BMI, and waist circumference were controlled in all analyses. HOMA-IR was calculated as fasting glucose (mg/dL)×fasting insulin (μIU/mL)/405. PMOS: polyendocrine metabolic ovarian syndrome; BMI: body mass index; TyG: triglyceride–glucose; TC: total cholesterol; HDL-C: high-density lipoprotein cholesterol; TG: triglycerides; HOMA-IR: homeostasis model assessment of insulin resistance; QUICKI: quantitative insulin sensitivity check index; FG/IR: fasting glucose-to-insulin ratio.

## DISCUSSION

In the present study, women with PMOS showed a distinct metabolic profile characterized by higher TyG index, HOMA-IR, and TyG-BMI values, together with lower QUICKI and FG/IR values, compared with healthy controls. These differences persisted after adjustment for age, BMI, and waist circumference, indicating that the observed metabolic differences were not explained solely by general or central adiposity. This finding is consistent with evidence showing increased long-term cardiometabolic risk in PCOS and tissue-specific insulin-signaling defects involving skeletal muscle, liver, adipose tissue, and ovarian steroidogenic pathways^11,12^.

The persistence of higher HOMA-IR and lower QUICKI and FG-IR values after adjustment supports IR as a central metabolic feature of PMOS. Previous reviews have emphasized that IR may occur across different tissues and may be present even without overt obesity^12^. Studies evaluating body composition and androgen excess also suggest that metabolic dysfunction in PMOS is not limited to BMI-defined obesity, and that visceral adiposity and hyperandrogenism may contribute to impaired glucose metabolism even in normal-weight women^13,14^. Thus, metabolic assessment in PMOS should not rely solely on BMI, but should include indices reflecting insulin sensitivity and cardiometabolic risk.

A key finding was that the TyG index remained significantly higher in the PMOS group after adjustment and was significantly associated with fasting-based surrogate measures of IR. This supports the potential of the TyG index as a complementary fasting lipid–glucose-based marker reflecting the metabolic profile associated with PMOS. Recent systematic reviews and meta-analyses have reported higher TyG and TyG-related indices in women with PCOS than in controls and associations with IR and adverse metabolic features^15,16^. Similar findings in broader populations suggest that TyG-related indices may provide useful complementary metabolic information, particularly where fasting insulin measurement is unavailable^17^. These findings should be interpreted as evidence of association rather than diagnostic accuracy and require further validation before clinical application.

TyG-BMI also remained significantly different between groups after adjustment. By combining a biochemical marker with an anthropometric measure, TyG-BMI may reflect both lipid–glucose dysregulation and adiposity-related metabolic burden. This is compatible with studies emphasizing the relevance of anthropometric and atherogenic indices in women with PCOS^18^. Nevertheless, the effect size of TyG-BMI in adjusted comparisons was lower than that of HOMA-IR and QUICKI. These findings suggest that TyG-BMI may provide complementary information regarding adiposity-related metabolic disturbances in PMOS, although its clinical utility requires further investigation.

The lipid-ratio findings require cautious interpretation. In unadjusted analyses, cholesterol/HDL and TG/HDL-C ratios differed between groups; however, after adjustment for age, BMI, and waist circumference, cholesterol/HDL lost statistical significance, whereas TG/HDL-C retained only a small effect size. This suggests that some lipid-ratio differences may be partly driven by anthropometric and age-related confounding. Previous studies have shown that lipid abnormalities in PMOS vary according to phenotype, adiposity, and androgen status^19,20^. The metabolic heterogeneity of PCOS has also been demonstrated longitudinally by Soares-Jr et al., who reported phenotype-related differences in metabolic syndrome and its components over six years of follow-up. These findings reinforce the importance of considering metabolic phenotype and adiposity when interpreting surrogate metabolic indices in women with PMOS^21^. Meta-analytic evidence also indicates that lipid ratios and TyG are altered in PCOS, although their magnitude and clinical interpretation may differ across populations and study designs^16^. Therefore, lipid ratios may provide supportive information, but their independent contribution appears more limited than that of TyG and insulin sensitivity indices.

The correlation analyses further support this interpretation. TyG was positively correlated with HOMA-IR and negatively correlated with QUICKI and FG-IR, and these associations persisted after adjustment for age, BMI, and waist circumference. This pattern indicates that TyG reflects a metabolic profile related to reduced insulin sensitivity. However, attenuation of some correlations after adjustment suggests that adiposity and central fat distribution contribute substantially to these relationships. This is clinically relevant because PMOS is heterogeneous, and metabolic risk may be shaped by interactions among IR, body composition, lipid abnormalities, and androgen excess^12-14^.

The relationship between IR indices and liver-related metabolic risk is also noteworthy. Although liver function parameters were not the primary focus of this analysis, previous studies in women with PCOS have demonstrated associations between IR indices and liver function markers, supporting the broader role of metabolic dysfunction beyond reproductive features^22^. This is particularly relevant because TyG and related lipid–glucose indices have been linked to hepatic steatosis and metabolic syndrome components in women with PCOS^9,16^. Accordingly, the metabolic abnormalities observed in our cohort may have implications for both IR and broader cardiometabolic and hepatic risk assessment.

The present study has several strengths, including the simultaneous evaluation of multiple fasting-based indices and adjustment for relevant anthropometric covariates. However, several limitations should be considered. First, the cross-sectional design precludes causal inference and does not permit assessment of the prospective predictive value of TyG-based indices. Second, IR was evaluated using fasting-based surrogate indices rather than direct methods of insulin sensitivity assessment, such as the hyperinsulinaemic–euglycaemic clamp; therefore, the observed relationships should not be interpreted as establishing diagnostic accuracy or clinically applicable thresholds. Third, the single-center design and the metabolic and anthropometric characteristics of the study population may limit generalizability. In addition, age-distribution balancing reduced the initially identified cohort and may have introduced selection bias despite the use of age as a covariate in adjusted analyses. Finally, residual confounding from unmeasured lifestyle, dietary, and body-composition factors cannot be excluded. Prospective multicenter studies incorporating direct measures of insulin sensitivity are needed to further clarify the clinical utility of TyG-based indices in PMOS.

## CONCLUSION

TyG and TyG-BMI were higher in women with PMOS than in healthy controls and were significantly associated with fasting-based surrogate measures of IR. These findings suggest that TyG-based indices may provide complementary information regarding the metabolic profile associated with PMOS. The present findings do not establish diagnostic accuracy or clinically applicable thresholds for IR. Prospective multicenter studies incorporating direct measures of insulin sensitivity are needed to determine the clinical utility of TyG-based indices in women with PMOS.

## Data Availability

The datasets generated and/or analyzed during the current study are available from the corresponding author upon reasonable request.
